# Corticosterone administration targeting a hypo-reactive HPA axis rescues a socially-avoidant phenotype in scarcity-adversity reared rats

**DOI:** 10.1016/j.dcn.2019.100716

**Published:** 2019-10-17

**Authors:** Rosemarie E. Perry, Millie Rincón-Cortés, Stephen H. Braren, Annie N. Brandes-Aitken, Maya Opendak, Gabriella Pollonini, Divija Chopra, C. Cybele Raver, Cristina M. Alberini, Clancy Blair, Regina M. Sullivan

**Affiliations:** a Department of Applied Psychology, New York University, 627 Broadway, New York, NY 10012, USA; b Emotional Brain Institute, Nathan Kline Institute & Department of Child and Adolescent Psychiatry, New York University School of Medicine, 1 Park Ave, New York, NY 10016, USA; c Center for Neural Science, New York University, 4 Washington Place, New York, NY 10003, USA

**Keywords:** Social behavior, Social avoidance, CORT, Corticosterone, Cortisol, Development, Prefrontal cortex, Glucocorticoid, Hippocampus, Stress, Early-life adversity, Poverty, Scarcity, Hypocorticosteronism, Hypocortisolism, HPA axis

## Abstract

It is well-established that children from low-income, under-resourced families are at increased risk of altered social development. However, the biological mechanisms by which poverty-related adversities can “get under the skin” to influence social behavior are poorly understood and cannot be easily ascertained using human research alone. This study utilized a rodent model of “scarcity-adversity,” which encompasses material resource deprivation (scarcity) and reduced caregiving quality (adversity), to explore how early-life scarcity-adversity causally influences social behavior *via* disruption of developing stress physiology. Results showed that early-life scarcity-adversity exposure increased social avoidance when offspring were tested in a social approach test in peri-adolescence. Furthermore, early-life scarcity-adversity led to blunted hypothalamic-pituitary-adrenal (HPA) axis activity as measured *via* adrenocorticotropic hormone (ACTH) and corticosterone (CORT) reactivity following the social approach test. Western blot analysis of brain tissue revealed that glucocorticoid receptor levels in the dorsal (but not ventral) hippocampus and medial prefrontal cortex were significantly elevated in scarcity-adversity reared rats following the social approach test. Finally, pharmacological repletion of CORT in scarcity-adversity reared peri-adolescents rescued social behavior. Our findings provide causal support that early-life scarcity-adversity exposure negatively impacts social development *via* a hypocorticosteronism-dependent mechanism, which can be targeted *via* CORT administration to rescue social behavior.

## 1 Introduction

Social skills are foundational to an individual’s success in school and life (Parker et al., 2006). The attainment of appropriate social skills facilitates learning and academic achievement (McClelland et al., 2000; Perry et al., 2018), as well as healthy relationships and an individual’s sense of belonging (Over, 2016). It is well-documented that socioeconomic disadvantage and its related adversities place children at higher risk for developing disrupted social behaviors, as evidenced by increased instances of social withdrawal (Zilanawala et al., 2019), reduced social skills (Bobbitt and Gershoff, 2016; Bulotsky-Shearer et al., 2008; Youngblade and Mulvihill, 1998; Zilanawala et al., 2019), and difficulty understanding and responding to social cues (Dumas et al., 2005). Whether socioeconomic disadvantage impacts social development appears to be determined, at least in part, by parenting quality. While parental warmth and sensitivity are protective in environments of socioeconomic disadvantage (Kirby et al., 2019), poverty-related adversities can at times encompass reduced sensitive caregiving. Indeed, high stress environments place parents at risk for less sensitive caregiving (Finegood et al., 2016; Perry et al., 2019). In turn, lower caregiving quality can mediate the effects of adversity on child outcomes, including social skills (Devenish et al., 2017). However, the biological mechanisms by which poverty-related adversities affect social development remain poorly understood and are difficult to explore using human research alone. Understanding how early-life poverty-related adversities might become biologically embedded to increase the risk of altered social development can ultimately inform more effective strategies for the prevention and remediation of disrupted social development (Shonkoff, 2010).

### 1.1 Early-life adversity, stress physiology, and social behavior

A likely mechanism by which early-life poverty-related adversities can biologically contribute to altered social development is *via* disruption of developing stress physiology. Exposure to adversity in early life, including poverty-related adversities, is consistently linked with disrupted hypothalamic-pituitary-adrenal (HPA) axis activity as measured *via* cortisol secretion in children (Bernard et al., 2015; Blair et al., 2013; Karb et al., 2012; Zalewski et al., 2012). However, research findings related to adversity and HPA axis function are mixed, with evidence of both elevated and blunted HPA axis activity following adversity exposure (for review see Doom and Gunnar, 2013; Dowd et al., 2009). A meta-analysis found robust evidence that the time elapsed since adversity onset helps explain these mixed and seemingly contradictory findings. Specifically, the authors found that time since adversity onset is negatively associated with HPA axis activity, such that as more time elapses the more a person’s cortisol secretion decreases (Miller et al., 2007). Indeed, it is widely theorized that although chronic adversity leads to heightened activation of the HPA axis, cortisol production downregulates as the HPA axis becomes overstrained over time–a process referred to as hypocortisolism (*e.g*., Fries et al., 2005; Heim et al., 2000). While prior studies have linked poverty-related adversity to reduced cortisol secretion in children and adults (Bernard et al., 2015; Karb et al., 2012; Zalewski et al., 2012), the subsequent consequences of hypocortisolism on social behavior remain poorly understood (for review see Charmandari et al., 2003; Gunnar and Vazquez, 2001; Miller, 2018; Raulo and Dantzer, 2018; van Goozen et al., 2007). Furthermore, animal models are needed to discern the causality/directionality of relations between adversity exposure, HPA axis activity, and social development, as well as to explicitly test these relations as a function of developmental timing.

Animal models are also needed to elucidate the effects of early-life adversity on neural regulation of the HPA axis, which remain far less understood and are much more difficult to probe in children. The HPA axis, which shows high levels of conservation across mammalian species, is regulated by a negative neuro-feedback circuit in the brain (Herman et al., 2016). Specifically, elevated CORT (cortisol in humans, corticosterone in rodents) typically suppresses HPA activation by occupying glucocorticoid receptors in the brain, which are normally abundant in the hippocampus and medial prefrontal cortex (mPFC) (Herman et al., 2016, 2012). Targeted studies indicate that glucocorticoid receptors in the hippocampus and mPFC are necessary for regulating basal levels of HPA axis activity, and selectively contribute to negative feedback of the HPA axis following exposure to an acute stressor (Furay et al., 2008; McKlveen et al., 2013; van Haarst et al., 1997). Furthermore, animal and human findings alike suggest that the hippocampus and mPFC are also important for social behavior (Bicks et al., 2015; Montagrin et al., 2018; Opendak et al., 2016; Rubin et al., 2014), and are particularly vulnerable to the long-term effects of early-life adversity (Andersen and Teicher, 2008; Gorka et al., 2014; Loi et al., 2014). Thus, biological alterations of the hippocampus and mPFC are candidate mediating links between poverty-related adversity exposure and altered social development.

Overall, there is strong evidence to suggest that poverty-related adversity in early life leads to increased HPA axis activity initially, followed by eventual downregulation of HPA axis activity over time. Thus, we theorize that disruptions of HPA axis activity and the HPA neuro-feedback loop mediate the effects of early-life poverty exposure on later social behavior. By testing this proposed mechanism, the current study aims to provide insight into neural and physiological processes which may be amenable to change *via* interventions.

### 1.2 The current study

The purpose of the current study was to use a rodent model of scarcity-adversity, which encompasses material resource deprivation (scarcity) and reduced caregiving quality (adversity) (Perry et al., 2019), to begin to probe causal mechanisms by which scarcity-adversity exposure in early life influences later-life HPA axis activity, glucocorticoid receptor levels in brain regions implicated in HPA axis neuro-feedback, and social behavior. To create conditions of scarcity-adversity, we utilized a “limited bedding” rodent protocol whereby a rodent dam was supplied with insufficient nest-building materials, so that she could not build an ideal nest for her pups (Perry and Sullivan, 2014; Raineki et al., 2010; Rincón-Cortés and Sullivan, 2016). This procedure causes the mother to spend less time interacting with her pups and induces rough transport of pups and stepping on pups (Perry et al., 2019). We assessed if this rodent model of scarcity-adversity would produce altered social behavior and HPA axis activity in peri-adolescence, a transitional time which encompasses the maturation of social behavior (Sisk and Foster, 2004) and is increasingly understood to be a sensitive period for programming of HPA function (for review see Holder and Blaustein, 2014; McCormick et al., 2017; McCormick and Mathews, 2007). Consistent with prior reports from our group (Raineki et al., 2012, 2015; Rincón-Cortés and Sullivan, 2016), we hypothesized that this rodent model of early-life scarcity-adversity would produce altered social behavior in peri-adolescence, as evidenced by reduced approach to a same-sex conspecific in a social approach test. We further hypothesized that the scarcity-adversity rearing effects on peri-adolescent social behavior would be mediated by blunted HPA axis activity as evidenced by reduced adrenocorticotropic hormone (ACTH) and CORT secretion, as well as upregulated glucocorticoid receptors in the hippocampus and mPFC. Thus, in Experiment 1, we tested the effects of early-life scarcity-adversity on later-life social behavior in a social approach test. We also tested the effects of early-life scarcity-adversity on HPA axis activity (ACTH and CORT reactivity) following the social approach test. Lastly, we tested the impact of early-life scarcity-adversity on glucocorticoid receptor levels in the hippocampus and mPFC, sites of CORT binding for the suppression of HPA activation. In Experiment 2, we pharmacologically increased CORT *via* intraperitoneal injections prior to the social approach test to assess if hypocorticosteronism causally mediated social behavior differences following scarcity-adversity rearing.

## 2 Materials and methods

### 2.1 Subjects

Male and female Long-Evans rats were bred and housed within an animal facility within the lab to ensure controlled rearing of all subjects. The day of birth was considered postnatal day (PN) 0, and litters were culled to 12 pups (6 males, 6 females) on PN1. Before and after our PN8-12 scarcity rearing manipulation (see “Early-Life Scarcity-Adversity Model” methods below), animals were housed with their mother in polypropylene cages (34 × 29 × 17 cm) with wood shavings materials used for nest building, and *ad libitum* food (Purina LabDiet #5001) and water. At PN23, animals were weaned from their mother and pair-housed with a sex- and age-matched cage mate in a polypropylene cage (34 × 29 × 17 cm), with access to ample wood shavings, and *ad libitum* food (Purina LabDiet #5001) and water. Animals were housed in a temperature (20 ± 1 °C) and light (12 h light/dark cycle) controlled room. Animals were tested in peri-adolescence (*e.g*., the time immediately prior to and during the onset of puberty, PN37-47), and each subject was only used once, with one male and one female used per litter per experimental group. All procedures were approved by the Institute’s Animal Care and Use Committee, which follow National Institutes of Health’s guidelines for the care and use of laboratory animals.

### 2.2 Early-life scarcity-adversity model

Litters were randomly assigned into scarcity-adversity or control rearing conditions on PN8. In control conditions, litters were provided with ample wood shavings, a material used by mothers to build a nest for her pups, which serves as the center of caregiving and a secure base for pups. Dams typically provide sensitive caregiving to their pups by spending frequent time in the nest nursing, licking, and grooming pups, as well as carefully retrieving pups that wander out of the nest (Perry et al., 2019). In scarcity-adversity conditions, litters were provided with insufficient nest-building materials (100 mL of wood shavings to create a thin layer covering the floor) so that the mother could not build a proper nest for her pups. This procedure causes the mother to spend less time interacting with and nursing pups, as she repeatedly attempts to build a nest, though pups gain weight normally (Perry et al., 2019). Furthermore, this scarcity-adversity model increases the time the mother spends roughly transporting pups and stepping on pups, which increases pups’ pain-associated vocalizations (Perry et al., 2019; Roth and Sullivan, 2005) and CORT (Raineki et al., 2010). Scarcity-adversity conditions persisted from pup age PN8-12. Pups were exposed to scarcity-adversity conditions on the morning of PN8, until the evening of PN12. The procedure has been used previously in our lab and others (Cui et al., 2006; Perry and Sullivan, 2014; Raineki et al., 2012, 2010; Raineki et al., 2015; Rincón-Cortés and Sullivan, 2016; Roth and Sullivan, 2005).

### 2.3 Social approach test

Social behavior was tested when subjects were PN37-47 (subject tested one time only) using a two-chamber apparatus (45.5 × 30.5  × 45 cm). The apparatus was constructed with black Plexiglas walls, a clear Plexiglas bottom, and a black Plexiglas division with a square opening (15  × 13 cm), which allowed animals to move between chambers. Each chamber contained a metal cube (6 × 6  × 6 cm) with circular holes (1 cm), which enabled animals to communicate *via* olfactory, tactile, and auditory cues but prevented sexual and/or aggressive social interactions (Ricceri et al., 2007). Before testing, the subject was acclimated in the apparatus for 5 min. Animals were excluded from testing if they failed to habituate to both chambers (spent less than 20% of time in either chamber of the apparatus). Following acclimation, the subject was removed from the apparatus and a younger (PN25-35), same-sex animal was placed in the metal cube in the social stimulus chamber, while the cube in the other chamber remained empty. The subject was then placed back into the apparatus, in the chamber without a social stimulus, and the time spent in each chamber was recorded for 10 min. All testing occurred during the light period (ZT3-ZT7, zeitgeber time, ZT0 represents light on, ZT12 represents light off). Testing was recorded using Ethovision software (Noldus, Leesburg, VA). Social behavior was quantified by time spent in each chamber of the apparatus, with decreased time spent in the chamber containing the social stimulus relative to the non-social chamber defined as social avoidance (Toth and Neumann, 2013). Number of chamber crossings was also measured as an index of general locomotor activity (Raineki et al., 2012; Rincón-Cortés and Sullivan, 2016). All behavior was manually scored from videos by an observer blind to the experimental conditions.

### 2.4 CORT and ACTH radioimmunoassay

Trunk blood samples were collected in 1.5 mL Eppendorf tubes (Hauppauge, NY) immediately following completion of the social approach test (or before the test for baseline comparison littermates). Baseline samples were collected from littermates who did not undergo behavioral testing. The samples were centrifuged at 3000 rpm (Fisher Scientific accuSpin model Micro17R Microcentrifuge, Waltham, MA) at 4 °C for 15 min to separate red blood cells from the serum. Aliquotted serum samples were stored at −80 °C until time of radioimmunoassay analysis. Samples were analyzed in duplicate for CORT levels using MP Biomedical Corticosterone Double Antibody RIA kit (Santa Ana, CA) and a gamma counter. ACTH levels were analyzed from samples in duplicate by commercial assay services (AssayGate, Inc., Ijamsville, MD).

### 2.5 Western blot analysis

Western blot analysis was carried out as previously described (Chen et al., 2012). Subjects were euthanized and brains removed 20 h following the social approach test at PN37-47 to survey molecular changes in response to the social approach test. Brains were stored in a −80 °C freezer prior to being mounted on a cryostat from which the mPFC, dorsal hippocampus, and ventral hippocampus punches were done with a neuro punch (19 gauge; Fine Science Tools, Foster City, CA). The punched tissue was then homogenized in ice-cold RIPA buffer (50 mM Tris base, 150 mM NaCl, 0.1% SDS, 0.5% Na-Deoxycholate, 1% NP-40) containing protease and phosphatase inhibitors (commercial protease and phosphatase inhibitor cocktails [Sigma Aldrich, St. Louis, MO] and 0.5 mM PMSF, 2 mM DTT, 1 mM EGTA, 2 mM NaF, 1μM microcystine, 1 mM benzamidine, 1 mM sodium orthovanadate). Bio-Rad protein assay (Bio-Rad Laboratories, Hercules, CA) was used to determine protein concentration. Equal amounts of total protein (*e.g*., 20 μg per lane) per sample was resolved on Criterion TGX Precast Gels (Bio-Rad Laboratories, Hercules, CA) and transferred to Bio-Rad Trans-Blot Turbo Midi Format PVDF transfer membranes (Bio-Rad Laboratories, Hercules, CA). Membranes were then dried and reactivated in methanol and washed with water before being incubated with the blocking solution, Odyssey Blocking Buffer (LI-COR Biosciences, Lincoln, NE), for 1 h at room temperature. Membranes were then incubated overnight with primary antibodies at 4 °C in solution per manufacturer's suggestion. Primary antibodies included anti-glucocorticoid receptor (1:200, cat # PA1-512, Thermo Fisher Scientific, Waltham, MA) and anti-actin antibody (1:10,000, Santa Cruz Biotechnology, cat# sc-4778, Dallas, TX), which was used to co-stain all membranes. On the following day, membranes were washed in TBS with 0.2% Tween20 (TBST) and incubated with a goat anti-rabbit IRDye 800CW (1:10,000; for anti-glucocorticoid receptor) and goat anti-mouse IRDye 680 L T (1:10,000; for anti-actin) from LI-COR Bioscience (Lincoln, NE) for 1 h at room temperature. Membranes were then washed again in TBST and scanned using the Odyssey Infrared Imaging system (LI-COR Bioscience, Lincoln, NE). Data were quantified using pixel intensities within Odyssey software (Image Studio 4.0) according to the manufacturer’s protocol (LI-COR Bioscience, Lincoln, NE). Relative quantification of actin was used for normalization (loading control).

### 2.6 Experiment 1: scarcity-adversity impacts on social behavior and HPA axis activity

**Fig. 1:**
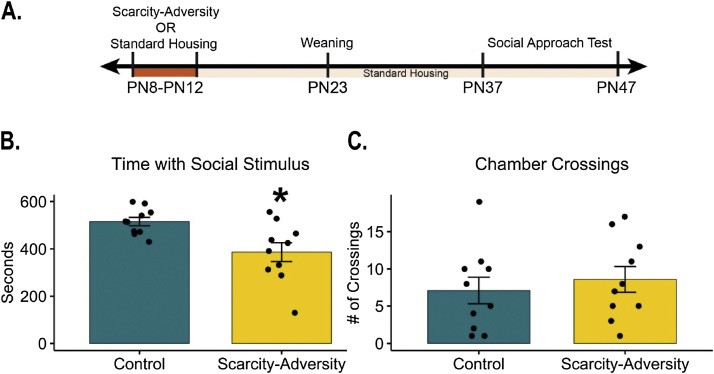
**Early-life scarcity-adversity reared peri-adolescent rats showed decreased time with a social stimulus rat in the social approach test**. **(A)** Experimental timeline. **(B)** Mean (±SEM) time spent in the social stimulus chamber during the social approach test (*significant difference between groups, *p* < 0.05, *n* = 10/group). **(C)** Mean (±SEM) number of crossings between social stimulus chamber and empty chamber during the social approach test (*n* = 10/group).

**Fig. 2:**
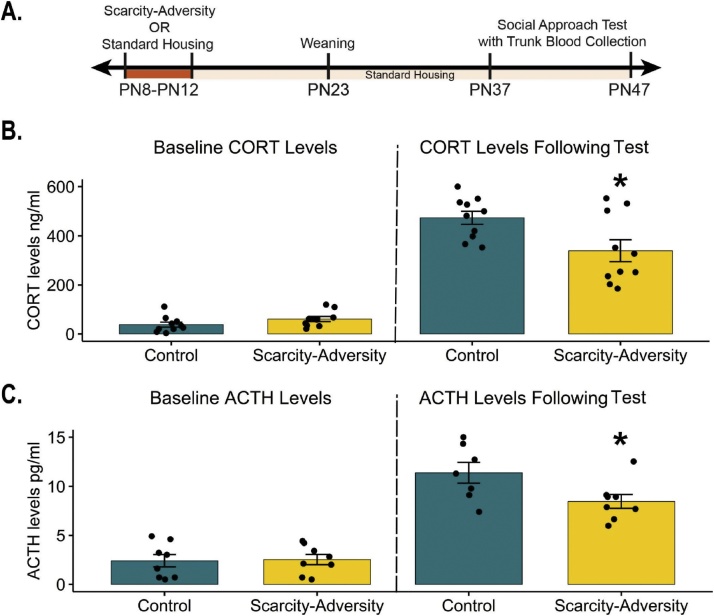
**Early-life scarcity-adversity rearing produced blunted corticosterone (CORT) and adrenocorticotropic hormone (ACTH) reactivity to the social approach test in peri-adolescence. (A)** Experimental timeline. **(B)** Mean (±SEM) levels of CORT assessed in animals that did not undergo behavioral testing, and in littermates following completion of the social approach test (*significant difference between groups, *p* <  0.05, *n* = 10/group). **(C)** Mean (±SEM) levels of ACTH assessed in animals that did not undergo behavioral testing, and in littermates following completion of the social approach test (*significant difference between groups, *p* <  0.05, *n* = 7–8/group).

**Fig. 3:**
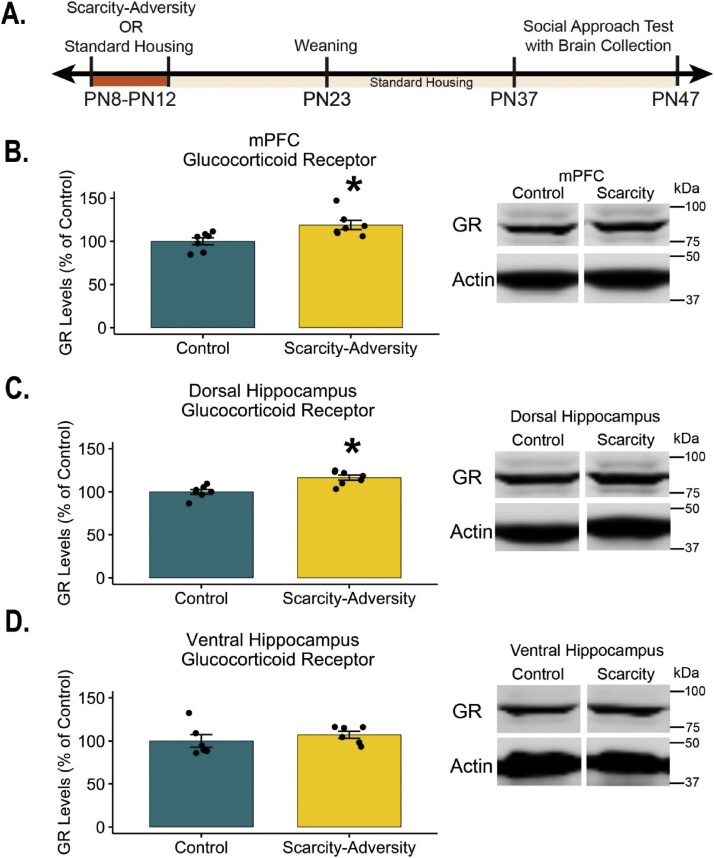
**Early-life adversity rearing increased glucocorticoid receptors levels in the medial prefrontal cortex (mPFC) and dorsal hippocampus following the social approach test in peri-adolescence. (A)** Experimental timeline. **(B)** Mean (±SEM) percent of glucocorticoid receptor (GR) levels in the medial prefrontal cortex (mPFC), normalized to control levels (*significant difference between groups, *p* <  0.05, *n* = 7/group). **(C)** Mean (±SEM) percent levels of glucocorticoid receptor (GR) levels in the dorsal hippocampus, normalized to control levels (*significant difference between groups, *p* <  0.05, *n* = 7/group). **(D)** Mean (±SEM) percent levels of glucocorticoid receptor (GR) levels in the ventral hippocampus, normalized to control levels (*p* =  0.403, *n* = 6/group).

Following early-life rearing conditions, control and scarcity-adversity animals were tested in the social approach test in peri-adolescence (PN37-47; Fig. 1A). Subjects were sacrificed either immediately following completion of the social approach test and trunk blood was collected for CORT/ACTH assay (Fig. 2A), or 20 h following the social approach test and brains collected for western blot analysis (Fig. 3A). Trunk blood was also collected from littermates who did not undergo behavioral testing to assay for baseline CORT levels. All testing and specimen collection occurred during the light phase (ZT3-ZT7).

### 2.7 Experiment 2: corticosterone administration

**Fig. 4:**
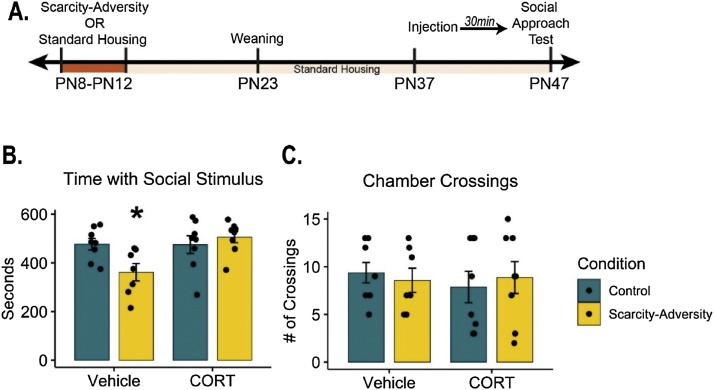
**Corticosterone injection rescues scarcity-adversity induced social behavior in social approach test during peri-adolescence. (A)** Experimental timeline. **(B)** Mean time spent in the social stimulus chamber during the social approach test following intraperitoneal injections of vehicle (saline) or CORT. Pharmacologically increasing CORT prior to social behavior testing normalized the amount of time that scarcity-adversity reared rodents spent with a social stimulus (*significantly different from all other groups, *p* <  0.05, *n* = 7–8/group). **(C)** Mean (±SEM) number of crossings between social stimulus chamber and empty chamber during the social approach test (*n* = 7–8/group).

Following early-life rearing conditions, scarcity-adversity and control animals were tested in the social approach test in peri-adolescence (PN37-47). Thirty minutes prior to social behavior testing, animals were injected intraperitoneally with either CORT (20 mg/kg, mixed in sterile saline; Sigma-Aldrich, St. Louis, MO) or vehicle (sterile saline) (Fig. 4A). Thus, Experiment 2 tested if pharmacologically increasing CORT could normalize scarcity-adversity subjects’ social approach levels in the social approach test.

### 2.8 Statistical analysis

All data were analyzed using Prism 7 (Graphpad Software Inc., San Diego, CA). The data were analyzed using Student’s *t* tests for paired comparisons, or by two-way analysis of variance (ANOVA) followed by *post hoc* Fisher tests between groups. All analyses were two-tailed. The significance of the results was accepted at *p* < 0.05. *A priori* power analyses indicated that a minimum final group size of six rats was required to have a probability of detecting significant group effects for behavior and biochemistry experiments. For biochemical studies, power calculation of *t* tests comparing early-life experience groups analyzed by G*Power software indicated a minimum sample size of six rats per group was necessary to achieve power of 0.8 and an error probability of 0.05. For behavioral experiments, similar power analyses calculated the requirement of a minimum sample size of six for two-way ANOVA to achieve power of 0.8 and an error probability of 0.05. To ensure a final sample size of six rats per group for each experiment, ten animals per group were behaviorally tested and/or had samples extracted (*e.g*., brain tissue, blood). Blood was excluded from processing if the minimal serum volume (160 μL) needed for processing was not obtained. This led to the exclusion of four samples (two controls, two scarcity-adversity) for baseline ACTH assessment and five samples (three controls, two scarcity-adversity) for ACTH assessment following the social approach test. No samples were excluded from CORT assessment. Brain tissue was excluded from immunohistochemistry processing if tissue was not extracted and frozen in under 60 s, to ensure the integrity of our proteins of interest, leading to the exclusion of six samples (three controls, three scarcity-adversity). Two additional samples of the ventral hippocampus were excluded from final analyses due to technical problems related to gel resolution which prevented the quantification of two bands. All data were checked for statistical outliers using a ROUT outlier test with Q = 1% prior to analyses. No significant outliers were identified. Statistical tests were designed using the assumption of normal distribution and variance for control *vs.* scarcity-adversity groups.

## 3 Results

### 3.1 Experiment 1: scarcity-adversity impacts on social behavior and HPA axis activity

Early-life scarcity-adversity rearing produced a significant disruption in social behavior in peri-adolescent (PN37-47) rats. Specifically, results of a *t* test revealed that scarcity-adversity reared peri-adolescent rats spent decreased time in the chamber of the social approach testing apparatus that contained a same-sex social stimulus animal, relative to control reared rats (Fig. 1**B**; *t*_(18)_ = 2.953, *p* = 0.009). An additional *t* test did not indicate a significant effect of early-life experience on the average number of crossings made during the social approach test (Fig. 1**C**; *t*_(18)_ = 0.603, *p* = 0.554). This suggests that the observed effect of early-life scarcity-adversity is not due to between-group differences in locomotion.

Assessment of trunk blood in peri-adolescent rats following completion of the social approach test revealed lower HPA axis activity, as indicated by reduced circulating CORT (Fig. 2**B**; *t*_(18)_ = 2.580, *p* = 0.019) and ACTH (Fig. 2**C**; *t*_(13)_ = 2.348, *p* = 0.035) in scarcity-adversity exposed subjects relative to controls. These CORT and ACTH differences were specific to HPA axis activity during the social approach test, as there was not a group difference in baseline CORT levels (Fig. 2**B**; *t*_(18)_ = 1.579, *p* = 0.132) or baseline ACTH levels (Fig. 2**C**; *t*_(14)_ = 0.138, *p* = 0.892) for littermates that did not undergo the social approach test.

Relative quantification using western blot analyses indicated significantly higher glucocorticoid receptors in the mPFC (Fig. 3**B**; *t*_(12)_ = 2.844, *p* = 0.015, *t* test) and dorsal hippocampus (Fig. 3**C**; *t*_(12)_ = 4.065, *p* = 0.002, *t* test) 20 h after the social approach test for scarcity-adversity reared compared to control reared peri-adolescents. However, no significant effect of early-life scarcity-adversity experience on glucocorticoid receptors was found in the ventral hippocampus 20 h after the social approach test (Fig. 3**D**; *t*_(10)_ = 0.873, *p*  = 0.403, *t* test). Taken together, findings from Experiment 1 suggest that early-life scarcity-adversity produces blunted HPA axis reactivity in a social approach test in peri-adolescence.

### 3.2 Experiment 2: corticosterone administration

Based on Experiment 1’s evidence of reduced HPA axis reactivity to the social approach test for scarcity-adversity reared subjects, Experiment 2 tested if pharmacologically increasing CORT could normalize scarcity-adversity subjects’ social approach. Results of a 2 × 2 ANOVA indicated a significant interaction of drug (CORT *vs*. vehicle) and early-life experience (scarcity-adversity *vs*. control) on time spent with a social stimulus rat (Fig. 4**B**; *F*_(1,27)_ = 5.861, *p* =  0.023). *Post hoc* tests indicated that intraperitoneal injections of CORT 30 min prior to testing normalized the time scarcity-adversity reared animals spent with a social stimulus rat in the social approach test (*p* < 0.05). CORT injections at this dose did not significantly affect social approach levels in control reared rats (*post hoc* tests, *p* < 0.05). Furthermore, a 2 × 2 ANOVA revealed no significant interaction of drug and early-life experience on the mean number of chamber crossings in the social approach test (Fig. 4**C**; *F*_(1,27)_ = 0.386, *p* =  0.540).

## 4 Discussion

Exposure to scarcity-related adversities during early life is associated with altered social functioning in humans (Bobbitt and Gershoff, 2016; Youngblade and Mulvihill, 1998; Zilanawala et al., 2019) and rodents (Raineki et al., 2015; Rincón-Cortés and Sullivan, 2016); however, the mechanisms mediating this association remain unclear. Here, we demonstrate that scarcity-adversity, as modeled by reducing nest building materials to induce rough and neglectful maternal behaviors, produces a socially-avoidant phenotype in peri-adolescence. These data are consistent with prior findings that early-life scarcity-adversity induces social avoidance in juvenile and adolescent rats (Raineki et al., 2012), but provide novel evidence regarding the mediating role of HPA axis activity. Furthermore, these data demonstrate for the first time that corticosterone administration can rescue the negative impact of scarcity-adversity rearing on social behavior.

Using our rodent model of scarcity-adversity, we were able to overcome limitations that we face when conducting human poverty research. Such limitations include the ability to randomly assign subjects into scarcity-adversity *vs.* control conditions and the ability to go beyond the assessment of neural and physiological correlates of behavior by directly testing underlying mechanisms (Perry et al., 2019). While our rodent model of scarcity-adversity does not encompass the complexity of human poverty, it provides complementary mechanistic evidence regarding how adverse experiences, such as scarcity-induced adverse caregiving behaviors, can become biologically embedded and in turn impact social development. Mechanistic research is ultimately needed to inform maximally-effective, targeted, and evidence-based interventions and policies for at-risk families and children (Wight et al., 2016).

The present study’s findings supported our hypothesis that early-life scarcity-adversity would produce reduced social behavior *via* a hypocorticosteronism-dependent mechanism. Specifically, Experiment 1 provided evidence that early-life scarcity-adversity rearing reduced later-life social behavior, as well as blunted HPA axis reactivity (circulating ACTH and CORT) to the social approach test. Our findings using a rodent model of scarcity-adversity are consistent with a broader human and animal literature supporting that early-life scarcity-related adversity can negatively impact social behavior outcomes (Brooks-Gunn and Duncan, 1997; Hosokawa and Katsura, 2017; Perry et al., 2018; Raineki et al., 2012; Rincón-Cortés and Sullivan, 2016; Zilanawala et al., 2019). Interestingly, our rodent model provided evidence that scarcity-adversity exposure confined to early life produced differences in later-life social behavior levels. That is, returning rodent mothers and her pups to normal bedding conditions by PN13 did not buffer offspring from the negative impacts of scarcity-adversity in peri-adolescence. This finding suggests that infancy (PN8-12) may be a foundational, sensitive period for social development. By leveraging the experimental control of a rodent model of scarcity-adversity, future research can further investigate how distinct developmental time windows of scarcity-adversity exposure might uniquely influence trajectories of social development.

Our findings of blunted HPA axis reactivity following scarcity-adversity exposure are consistent with existing theory (based on an extant human and animal literature) that extended exposure to stress can overstrain the HPA axis, resulting in subsequent downregulated CORT production (Fries et al., 2005; Heim et al., 2000; Miller et al., 2007). Specifically, it is possible that prolonged hypersecretion of CORT can produce tissue damage and/or dysregulation of biological systems important for HPA axis activity and regulation, such that CORT production eventually declines (Miller et al., 2007). Thus, hypocortisolism/hypocorticosteronism may occur as a protective response to chronic overactivation of the HPA axis by reducing the damaging effects of CORT, although this seemingly comes at the expense of behavior such as decreased social behavior. Our assessment of glucocorticoid receptor levels in the mPFC and hippocampus is in line with the suggestion of blunted HPA axis reactivity during the social approach test for scarcity-adversity reared subjects. Specifically, scarcity-adversity reared subjects displayed elevated expression of glucocorticoid receptors in the mPFC and dorsal hippocampus 20 h after completion of the social approach test, relative to control reared subjects. We speculate based on neuropharmacological principles of receptor expression that such an upregulation of glucocorticoid receptor levels might have been consequential to subjects’ blunted CORT output in response to the social approach test. Indeed, glucocorticoid receptor expression is directly influenced by CORT exposure, such that cells downregulate or upregulate expression levels in the presence of high or low levels of CORT, respectively (Miller et al., 2007). Moreover, it has been previously reported that glucocorticoid receptor levels are increased in the hippocampus and prefrontal cortex following high or chronic stress (Finsterwald et al., 2015; Guidotti et al., 2013).

CORT binding to glucocorticoid receptors in the brain, including in the mPFC and hippocampus, is vital to initiating the negative neuro-feedback circuit for neural regulation of HPA axis activity (Herman et al., 2016). Thus, upregulation of glucocorticoid receptors might be reflective of a compensatory response to insufficient circulating CORT during the social approach test, to improve neural regulation of HPA axis activity under subsequent behavioral conditions. That is, by increasing glucocorticoid receptor levels in response to low circulating CORT, the organism increases future chances of CORT binding to glucocorticoid receptors in the mPFC and hippocampus to initiate the negative neuro-feedback circuit. Alternatively, scarcity-adversity related differences in glucocorticoid concentrations and receptor levels could be due to developmental programming differences, resulting from bidirectional interactions between HPA axis activity and glucocorticoid concentrations in the mPFC and dorsal hippocampus during and following adversity. Thus, further experimental testing is needed to substantiate our purported compensatory mechanism, as well as to determine if such a compensatory mechanism can be leveraged to improve behavioral outcomes when chronic adversity is no longer present in the environment.

It is of note that we found upregulation of glucocorticoid receptor levels in the dorsal region, but not ventral region, of the hippocampus following the social approach test for scarcity-adversity reared rats. This was an unexpected result, as the ventral hippocampus has been previously implied in stress regulation (Fanselow and Dong, 2010). Both the hippocampus (including dorsal and ventral components) and mPFC send glutamatergic projections to GABAergic neurons in the paraventricular nucleus (PVN) of the hypothalamus to inhibit PVN activation, providing neural regulation of the HPA axis (Herman et al., 2016, 2012). Region-specific differences in glucocorticoid receptor expression following scarcity-adversity rearing may reflect altered developmental trajectories of the dorsal and ventral components of the hippocampus, which may be differentially impacted as a function of the developmental timing of scarcity-adversity exposure. Indeed, prior research has demonstrated that chronic stress differentially impacts the structure of dorsal and ventral components of the hippocampus (Czeh et al., 2015; Pinto et al., 2015). Further research is needed to disentangle unique windows of vulnerability for these developing brain regions, as well as the distinct developmental effects of altered glucocorticoid receptor levels in these brain regions.

Importantly, our rodent findings of blunted HPA axis reactivity following scarcity-adversity rearing mirror human findings relating early adversity exposure to HPA axis activity. For example, prior research has reported that children with a history of early-life adversity display blunted cortisol activity, which in turn is associated with increased reporting of child behavioral problems (Alink et al., 2012; Bernard et al., 2015). Furthermore, accumulating evidence suggests that poverty and its related adversities are associated with a downregulation of HPA axis activity after chronic periods of elevated stress responding (Badanes et al., 2011; Dulin-Keita et al., 2012; Zalewski et al., 2012). Thus, our rodent model of scarcity-adversity shows promise regarding its translational validity in relation to the impact of scarcity-adversity on social behavior and HPA axis development. Furthermore, our rodent model allowed us to directly test if hypocorticosteronism following scarcity-adversity rearing underlies reduced social behavior in peri-adolescence.

Results from Experiment 2 provided causal support for our hypothesis that scarcity-adversity induced hypocorticosteronism would mechanistically underlie reduced social behavior levels. Specifically, intraperitoneal CORT injections prior to the social approach test was sufficient in normalizing scarcity-adversity reared subjects’ social behavior levels relative to control reared subjects. This finding highlights a direct link between circulating CORT levels and social behavior, and converges with prior human research reporting that hypocortisolism mediates relations between early-life adversity and later-life social behavior (Koss et al., 2016; Pitula et al., 2017). Additionally, CORT-induced normalization of social behavior provides support to the notion that moderate levels of stress arousal can benefit behavioral and cognitive performance (Arnsten and Li, 2005). Indeed, both atypically high *and* low CORT reactivity profiles are associated with an inflexible, dysregulated stress response system (Blair et al., 2011; Bruce et al., 2009).

### 4.1 Implications for intervention, future directions, and limitations

The present study provides important implications regarding scarcity-adversity induced physiological processes which may be amenable to change *via* interventions. Specifically, moderately increasing circulating CORT levels in individuals with early-adversity induced blunted CORT may improve social behavior and developmental outcomes. The benefits of CORT administration on developmental outcomes may not be confined to social behavior outcomes. For example, we have previously demonstrated that systemic CORT administration rescues early-life adversity induced learning, hippocampal, and amygdalar deficits in rodent adults (Moriceau et al., 2009). Additionally, a recent study of women with a history of severe early-life adversity indicated that oral administration of CORT (*vs.* placebo administration) reduced depression-related negative memory bias for individuals with a history of severe early-life adversity (Abercrombie et al., 2018). Future experiments utilizing our rodent model of scarcity-adversity should test if the benefits of CORT administration are specific to social avoidance or generalizable to these related outcomes, such as learning and memory performance or depressive/anxiety-like behavior.

The present findings should be considered alongside the following limitations. While our rodent model of scarcity-adversity appears to hold some translational validity regarding developmental impacts of scarcity-adversity on social and HPA axis development, our model does not encompass the complex condition of human poverty. Furthermore, while our findings support a causal link between scarcity-adversity and reduced social behavior *via* hypocorticosteronism, further research aimed at replicating and expanding upon these findings is warranted. For example, future experiments should explore how blunted HPA axis reactivity occurs in scarcity-adversity reared animals using additional tests of social behavior (*e.g*., social interaction in an open arena, resident intruder test, social recognition test). Additionally, future experiments should explore if blunted HPA axis reactivity following scarcity-adversity rearing is specific to 1) tests of social behavior, 2) developmental timing of scarcity-adversity exposure, and 3) developmental timing of behavioral outcome testing. Furthermore, future experiments are warranted to disentangle if differences in glucocorticoid receptor distribution emerge during or following scarcity-adversity exposure, or whether they reflect changes induced by social behavior testing. The impact of long-term CORT administration and/or the ability to leverage short-term CORT administration for long-term rescue of developmental outcomes should also be examined. For example, future experiments are needed to determine the developmental time course and endurance of changes in glucocorticoid receptors and HPA axis markers following scarcity-adversity exposure, as well as how long-lasting the positive effects of CORT administration are. The high internal validity of our rodent model of scarcity-adversity well-positions us to answer such research questions.

### 4.2 Conclusion

In conclusion, the present study provides novel, causal evidence that early-life scarcity-adversity experience can get under the skin to alter later-life social behavior *via* a hypo-reactive HPA axis. Moreover, corticosterone administration targeting this hypo-reactive HPA axis can be used to rescue the social avoidant phenotype induced by early-life scarcity-adversity. Further research using animal models of scarcity-adversity will provide an increased understanding of potential sensitive periods of scarcity-adversity induced impacts on social development, as well as opportunities and mechanistically-informed strategies for prevention and interventions. Understanding methods by which to promote the attainment of appropriate social skills for at-risk children has the potential to improve children’s development across many areas of functioning, including social, emotional, and cognitive development. In light of the present results, policies aimed at scarcity-adversity/poverty reduction could be one such way to positively benefit social development. Furthermore, preclinical research that is translationally sensitive will improve chances of creating impactful preventative and interventional efforts for the improvement of both social behavioral outcomes and underlying neural and physiological processes.

## Declaration of Competing Interest

The authors declare that they have no known competing financial interests or personal relationships that could have appeared to influence the work reported in this paper.
